# Contour identical implants to bridge mandibular continuity defects - individually generated by LaserCUSING^®^ - A feasibility study in animal cadavers

**DOI:** 10.1186/s13005-016-0114-0

**Published:** 2016-04-11

**Authors:** Bernd Reitemeier, Christine Schöne, Raoul Lesche, Günter Lauer, Matthias C. Schulz, Jutta Markwardt

**Affiliations:** Department of Prosthetic Dentistry, Faculty of Medicine “Carl Gustav Carus”, Technische Universität Dresden, Fetscherstr. 74, 01307 Dresden, Germany; Department of Design Engineering/CAD, Institute of Machine Elements and Machine, Technische Universität Dresden, George-Bähr-Str. 3c, 01069 Dresden, Germany; H&E Produktentwicklung GmbH, Hofmann und Engel, Kunzer Marktweg 13, 01468 Moritzburg, Germany; Department of Oral and Maxillofacial Surgery, Faculty of Medicine “Carl Gustav Carus”, Technische Universität Dresden, Fetscherstraße 74, 01307 Dresden, Germany

**Keywords:** Cadaver study, CAD/CAM procedure, Continuity defects, Individual implant, LaserCUSING^®^

## Abstract

**Background:**

Ablative tumor surgery often results in continuity defects of the mandible. When an immediate reconstruction using autologous bone grafts is not possible the bridging of the defects with a variety of bridging plates might be achieved. However, those bridging plates have the risk of plate fractures or exposure. Customized titanium implants manufactured using CAD/CAM and the LaserCUSING^®^ technique might be an alternative.

**Methods:**

In the present study, computed tomographies (CT) of porcine cadaver mandibles were generated and transferred into DICOM data. Following, different continuity defects were surgically created in the mandibles. Based on the DICOM data customized titanium implants were manufactured using CAD/CAM procedures and the LaserCUSING^®^ technique. The implants were fixed to the remaining stumps with screws. Subsequently, the accuracy of the reconstructed mandibles was tested using plaster casts.

**Results:**

The workflow from the CT to the application of the customized implants was proved to be practicable. Furthermore, a stable fixation of the customized implant to the remaining stumps could be achieved. The control of the accuracy showed no frictions or obstacles.

**Conclusion:**

The customized titanium implant seems to be a promising approach to bridge continuity defects of the mandible whenever an immediate reconstruction with autologous bone is not possible.

## Background

Ablative tumor therapy is one of the most frequent reasons for continuity defects of the mandible [1]. Currently, the immediate reconstruction of the resulting defects using musculo-osseous flaps is considered as the most reliable therapy [2]. These flaps might be harvested from the iliac crest, the fibula or the scapula and are microvascularly anastomosed. However, the immediate reconstruction of mandibular continuity defects is not always possible. In those cases, a bridging of the defects using metallic plates is crucial to ensure the correct position of the remaining stumps and thus, the possibility of ingestion as well as the patency of the upper airways. By using reconstruction plates various complications have been described e.g. plate fractures, loosening of hardware or exposure of the metallic plate [3–5]. Furthermore, it is difficult to adapt the reconstruction plates to the different contours of the bone being normally achieved by intra-operative bending. This bending might result in weak points leading to a higher risk of plate fractures [6]. More satisfying results have been achieved by the pre-bending of reconstruction plates on molded medical rapid prototyping (MRP) models [7]. However, a form resembling the complex shape of the mandible could not be reached. The incongruity between the plate and the contour of the remaining mandible is prone to areas of dead space between the bone and the surrounding soft tissue [8]. This lack of soft tissue might result in a higher tension and thus, lead to plate exposure. Therefore, a customized implant being of the same contour as the resected region would be desirable. Furthermore, such an individualized implant could be produced preoperatively, fixed to remaining stumps by a tube-in-tube like connection and thus, prevent intra-operative adaptation.

One approach to achieve such implants could be the manufacturing of custom made titanium trays combined with autologous bone [9]. In the mentioned animal study could be shown that the bridging of continuity defects was mechanically stable and even the reconstruction of the bony continuity was possible. Another group applied in two cases a custom made mesh tray consisting of raw particular hydroxyapatite and poly-l-lactide to restore the mandible [10]. Furthermore, the substitution of parts of the mandible has been performed using the computer aided design and manufacturing (CAD/CAM) technology [11]. In a case series, the missing parts of the mandible including the temporo-mandibular joint were reconstructed with the help of stereolithographic models of the skull.

Another approach is the manufacturing of a customized mandibular implant applying the LaserCUSING^®^ technology. By using this sintering method it is possible to produce highly complex three-dimensional structures such as the mandibular bone [12, 13]. The data required to reproduce the complex shape of the mandible might be obtained from a computed tomography scan which is performed routinely during the tumor staging. The maxillofacial surgeon is defining the resection planes with regard to the safety margins on a virtual three-dimensional model of the mandible. Without the intermediate step of a stereolithographic model the customized implant might be manufactured.

The current project describes the virtual creation and the manufacturing of a customized titanium implant to bridge continuity defects of the mandible. Thus, porcine cadaver mandibles were used to evaluate the practicability of this approach.

## Methods

According to Markwardt et al. [1], four typical localizations of defects resulting from ablative tumor surgery in the mandible are:

1. The defect including the segment from the area between canine/first premolar and the second/third molar on the ipsi lateral side. In this cadaver study, the left side was used.
2. The defect extending between the central incisors and the second/third molar. In the study, the defect was created on the left side.
3. The defect extending from between the canine/first premolar to the second/third molar on the contra lateral side exceeding the midline.
4. The defect including the anterior segment of the mandible between canine/first premolar on both sides. In particular, this type of defect contained the chin area.

**Fig. 1:**
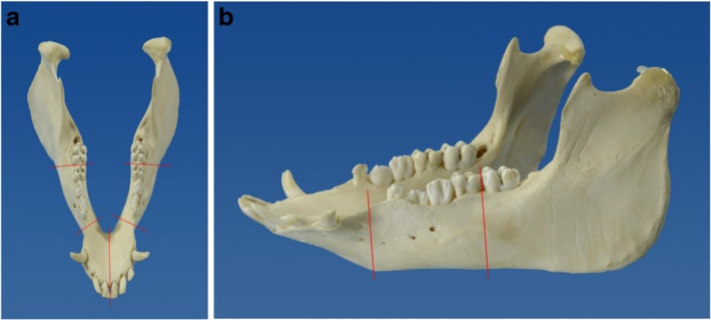
Typical defect localization resulting from ablative tumor surgery. **a** the numbers correlating with the defect localizations from a cranial perspective. **b** The resection planes of defect localization 1 in a lateral view

The different defect locations are shown in Fig. 1a, b.

The protocol of the study was authorized by the Commission for Animal Studies of the District Government Dresden, Germany (File No.: 21-9168.11-1/2010-25). Eight cadaver mandibles obtained from pigs, two for each defect localisation, were used. In order to assess the fitting of the customized implants to the mandibles plaster casts were made prior to performance of computed tomography and the resection. First, impressions of the pig mandibles were taken using a high precision addition cross linked impression silicone (Provil putty soft fast and Provil light C.D. 2 fast; Heraeus Kulzer, Hanau, Germany). The linear shrinkage during polymerization and the storage is less than 0.05 % [14]. Subsequently, pilot model casts were made using a standard modelling plaster with a maximal expansion of 0.14 % (Modelit blau, Siladent, Böhme & Schöps, Goslar, Germany).

### Computed tomography scan of the mandible and virtual design

In order to generate a data set of the contour of the mandible a thin-slice computed tomography (SOMATOM 16, Siemens Healthcare, Erlangen, Germany) was performed. This procedure is usually performed during the tumor staging. It is crucial to estimate the extent of the tumor and a potential infiltration of the mandibular bone. Subsequently, the data was transferred to digital imaging and communications in medicine (DICOM) files to a standard workstation. Using the software VoXim^®^ Osteo (IVS Technology GmbH, Chemnitz, Germany) a three-dimensional model of the mandible in surface tessellation language (STL) format was created.

**Fig. 2:**
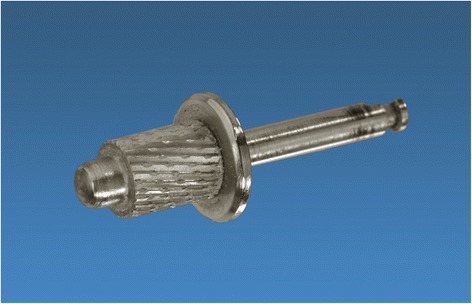
The newly developed step cutter to create the circular step in the mandibular stumps

On this virtual three-dimensional model the surgeon was defining the resection planes and thereby, determinating the part of the mandible required to be removed. The surgeon’s clinical and histological knowledge is crucial to allow the virtual resection of the tumor and the surrounding hard as well as soft tissue with appropriate safety margins. Hereby, the clinician has to pay attention to define the correct resection planes as far as special cutting aides were manufactured to ensure the correct position of the cutting planes during surgery. In order to allow a contour identical shape in the connection area between the customized implant and the stumps of the mandible this region was prepared as follows: A circular step of 1 mm with an extent of 6 mm was created in the remaining stumps. Therefore, a newly designed step cutter with a guiding mandrel was applied (Busch, Engelskirchen, Germany) (Fig. 2). Thus, the customized implant could be connected to the mandible like a tube-in-tube connection. In this overlapping connection area, each two holes were integrated in the implant on the lingual and buccal side to allow a fixation using screws. The virtual resection provided the information for the computer aided designing/computer aided manufacturing process of the individual implant. Subsequently, the customized implant was virtually created according to the region and the extent of the resulting defect.

### Manufacturing of the cutting aides and the customized implant

**Fig. 3:**
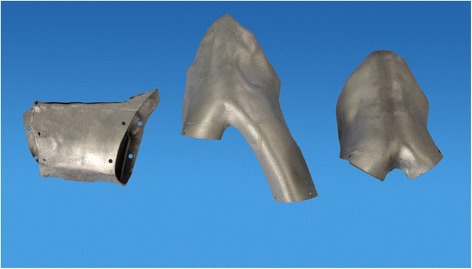
The customized implants manufactured by LaserCUSING® to bridge defect localization 1, 3 and 4 (from left to right)

The manufacturing of the cutting aides and the customized mandibular implant was performed by the industrial partner of the study (Hofmann und Engel Produktentwicklung GmbH, Moritzburg, Germany) using the LaserCUSING^®^ technology [15]. First, the area planned to be resected according to the virtually created three-dimensional model of the mandible was transformed into a set of STL data. Next, the customized implant was built in layers by laser sintering using pure titanium powder. The thickness of the layers varied from 30 μm to 50 μm. Thus, the manufacturing of highly complex geometrical structures is possible in an efficient amount of time. After cleaning in an ultrasound bath and sterilization the cutting aides and the customized implants were ready for application (Fig. 3).

### Model operations

**Fig. 4:**
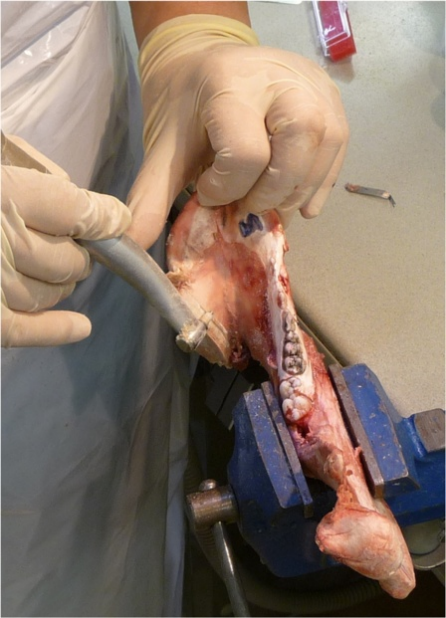
Preparing the circular mesial step to fix the customized implant (Cadaver operation)

**Fig. 5:**
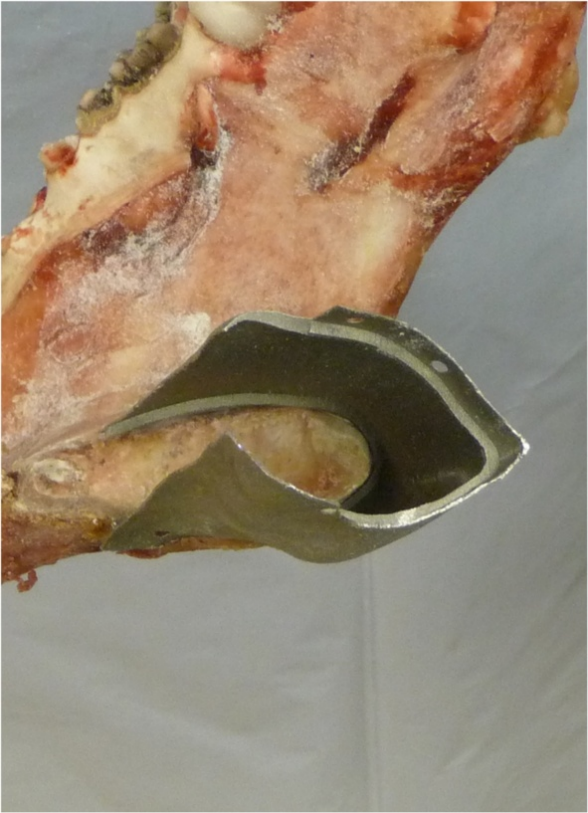
Detailed view into the connection area of the bone – implant area. A tight fit was achieved between all implants and the bone

To perform the model operations the mandibles were fixed in a bench vise (Fig. 4). Following, the complete procedure is described in detail for the defect localization number one:

In a first step, the cutting aides were fixed on the mandible. This step is crucial as the cutting planes had to correspond exactly to the virtually planned cutting planes. Next, the mandible was resected at the virtually determined cutting planes using a pad saw (Cayman 200 with pad saw type 68.24.10, Medicon Instruments, Tuttlingen, Germany). After the resection, the segment between the canine and the third molar was removed. Subsequently, the stumps were prepared to incorporate the customized implant. Therefore, the circular step of one millimetre depth and 6 mm extension was prepared on both stumps using a special designed step cutter with a guiding mandrel (Fig. 5) driven by a contra angle hand piece. Next, the customized implant was connected to both stumps in a way of a tube-in-tube connection. The implant was fixed to the mandibular bone by four self-cutting countersink screws (Ti Mini Easy Grip-screws 2.0 × 7.0 mm, Medicon Instruments, Tuttlingen, Germany) in the overlapping area of the implant and the stumps. Two screws were inserted either on the buccal and on the lingual side. On the lingual side, a right-angle drill and screwdriver were used. Those procedures were performed twice for each defect localization.

Following the model operations, the silicone impressions and plaster casts served to evaluate the correctness of the implant fitting. Therefore, the three-dimensional position of the remaining teeth was controlled regarding friction or free spaces between the silicone impression and the reconstructed mandible.

## Results

**Fig. 6:**
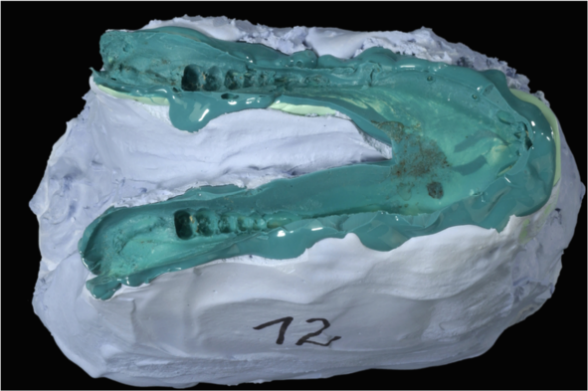
Plaster cast and impression of the cadaver mandible. The plaster was used to provide a framework for the elastic impression material

**Fig. 7:**
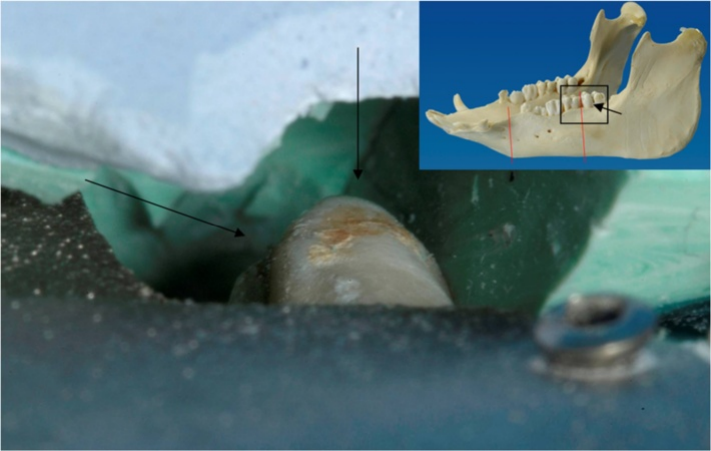
Reconstructed mandible (defect localization 1) on the control cast. In the upper part of the image the plaster cast (blue) and the silicone impression (green) are visible. The lower part shows a part of the customized titanium implant with one fixation screw. The tooth (molar) depicted in the center of the image is located posterior of the defect bridged by the customized implant. The silicone has been removed for control – a correct fit of the silicone to the tooth is obvious from various directions (arrows). This is comparable to the key-lock-principle. The perspective is as shown in the overview of the mandible (right upper corner)

The data collection using CT scans prior to ablative tumor surgery is a standard procedure during the preoperative staging. The creation of the three-dimensional model of the mandible was possible using a standard workstation. The definition of the cutting planes for the tumor resection including the safety margins could be easily performed by the surgeon on the virtual three-dimensional model of the mandible. No problems could be determined in transferring the data sets to the manufacturer. The LaserCUSING^®^ technology enabled a rapid and correct production of the cutting aides and the customized implants. It was possible to create the required devices and implants without a delay for a potential operation. The cutting aides could be easily fixed on the cadaver mandibles to ensure the correct position of the resection planes. Thus, it was possible to resect the pre-planned area of the mandible. An experienced surgeon was able to perform the resection using a micro pad saw and to prepare the remaining bone stumps with the step cutter. Following, the implants could be fitted to the created step on the mandibular stumps. No twisting or torsion occurred when connecting the implants to the stumps. A stable connection could be achieved even without the screw fixation. The drilling of the screw holes and the insertion of the four screws was possible by the methods used in a clinical situation. On the lingual site, the use of the right-angle drill and screwdriver seemed to be practicable. When setting back the reconstructed mandibles to the previously made silicone impressions a close congruency was observed (Fig. 6). No free spaces or frictions were found (Fig. 7).

## Discussion

An immediate and functional reconstruction of the mandible is crucial after continuity defects [2]. However, the primary reconstruction is not always possible or recommended considering the recurrence control or wound healing [16, 17]. Therefore, metallic plates are used to bridge the defect. The use of bridging plates has shown different shortcomings including the loosening of hardware and plate exposure [3–5]. Furthermore, if not pre-bent on a CAD/CAM model they have to be adapted during the surgical procedure. Thus, on the one hand the duration of the anaesthesia for the patient is extended. On the other hand, adapting the plates might cause weak points leading to a higher risk of plate fractures [6]. This risk of fracture might be overcome by the use of customized implants. No adaption of the implant or bending would be necessary. The mechanical properties of the customized implant have to be tested in biomechanical test to compare the stability to those of clinical established reconstruction plates. Compared to an untreated porcine cadaver mandible 47 % of the maximum force was necessary for the failure of the connection between bone and customized implant [18]. In another experimental study, it could be shown that a non-linear arrangement of the screws fixing the bridging plate might be advantageous [1]. A preoperative analysis of the bone thickness is favourable to find the optimal position of the fixation screws [19]. Furthermore, a shape resembling the original shape of the mandible could not be achieved by the use of bridging plates [20, 21]. Better aesthetic results have been obtained by pre-bending plates on molded models [7]. However, the incongruity between the plates and the remaining mandibular stumps might lead to higher tensions in the covering soft tissues and thus, increasing the risk of plate exposure [8]. A customized implant resembling the original shape of the mandible could improve these conditions. However, the risk of exposure of the titanium implant might not be eliminated totally as post-operative radiation is frequently applied after the resection of malignant tumors infiltrating bone. Therefore, one prerequisite though would be the sufficient covering by adequate soft tissues e. g. a radial forearm flap. On the one hand, titanium has a reduced scattered radiation when compared to other alloys [22]. Furthermore, the thickness of the customized implant is 0.3 mm, whereas clinically applied reconstructions plates are 3.0 mm in thickness. The reduced layer strength might reduce scattered radiation and thus, reduce additional damage to the adjacent soft tissue during therapeutic radiation. However, a first application to reconstruct a mandibular defect following trauma might be reasonable.

One advantage by using the approach described in the current study is the possibility to produce the customized implant pre-operatively. The data for the reconstruction is collected from computed tomography scans of the mandible. These scans are routinely performed during tumor staging and therefore, the data could be obtained without any timely delay or any further burden for the patient. In cases of bone deformation caused by the tumor the contra lateral side of the mandible might be mirrored to reconstruct the part needing to be resected. Like for any pre-fabricated customized implant a major drawback is the necessity to determine the resection planes pre-operatively. An intra-operative adaptation of the customized implant by the surgeon is not possible. Therefore, due to potential tumor infiltration into bone tissue the resulting safety margin will cause the necessity of an extensive resection of the bone to prevent tumor recurrence [17]. Here, the application of functional imaging e.g. positron emitting tomography/computed tomography could be beneficial to determine bone marrow invasion pre-operatively [23]. Thus, the risk of intra-operatively changes might be reduced. Furthermore, the time between imaging and resection should be kept as short as possible. This requirement can be achieved by the described method as it takes only 10 day between the computed tomography and the completion of the customized implant so that the operation might be performed promptly.

Another advantage of the technology is the possibility to shorten the process of manufacturing. Currently, high complex structures e.g. the mandibular bone are reconstructed using stereolithographic models [11]. By the application of the LaserCUSING^®^ technology only one single step of construction would be necessary. Thus, the production tolerance might be reduced. Furthermore, it has been shown that the surface of the implant produced by LaserCUSING^®^ is attracting osteoblast-like cells [24]. This might promote the ingrowths of bone cells into the implant and enable the osseointegration of the implant. Additionally, the customized implant is fixed in the way of a tube-in-tube like connection to the mandibular stumps. Thus, an enlarged area of bone-to-implant-contact compared to conventional bridging plates can be achieved. Furthermore, the screws securing the implant from mobility are located on the lingual and vestibular side in a cranial and caudal position. For biomechanical reasons, this position seems to be an advantage compared to the screw position longitudinally to the length axis of a conventional reconstruction plate [1]. Thus, a connection of higher stability compared to conventional bridging plates would be achieved. The insertion of the screws on the lingual side might arise problems in a clinical situation due to limited space. However, in the present cadaver study the screw insertion and fixation was possible using right-angle drills and screwdrivers.

## Conclusions

From the current results it can be concluded that the creation and manufacturing of a customized titanium implant to reconstruct continuity defects of the mandible is possible by LaserCUSING^®^ based on data obtained from computed tomography scans. In a next step, biocompatibility tests have to be performed in order to evaluate the potential practicability of this approach.
